# Tenosynovial giant cell tumor of the hip: a systematic review and institutional case series with Meta-analysis of recurrence and patient-reported outcomes

**DOI:** 10.1016/j.jbo.2026.100769

**Published:** 2026-05-25

**Authors:** E. van der Loos, D.T. Meijer, J.A.M. Bramer, H.W.B. Schreuder, F.G.M. Verspoor

**Affiliations:** a Department of Orthopaedic Surgery and Sports Medicine, Amsterdam UMC, University of Amsterdam, Meibergdreef 9, Amsterdam, the Netherlands; b Cancer Center Amsterdam, Amsterdam, the Netherlands; c Amsterdam Movement Sciences, Rehabilitation & Development, Amsterdam, the Netherlands; d Department of Orthopedics, Radboud University Medical Center, Nijmegen, the Netherlands.

**Keywords:** Tenosynovial giant cell tumor, Pigmented villonodular synovitis, Hip joint, Treatment outcomes, Recurrence rates, Systematic review, Patient-reported outcomes

## Abstract

**Background:**

Tenosynovial giant cell tumors (TGCT) are rare, locally aggressive neoplasms of the synovium, most frequently affecting the knee and less commonly the hip. Due to its deep anatomy and weight-bearing function, hip TGCT may follow a distinct clinical course. This study evaluates clinical presentation, treatment outcomes, recurrence patterns, and patient-reported outcomes in hip TGCT.

**Methods:**

A systematic literature search in PubMed, Embase, and Cochrane Library (January 2000–May 2025) identified studies including ≥5 patients with hip TGCT reporting recurrence, complications, functional outcomes or patient-reported outcomes. A random-effects meta-analysis assessed recurrence risk across treatment modalities. Additionally, 23 patients of two retrospective institutional cohorts were analyzed, including PROMIS-CAT assessment.

**Results:**

Seventeen studies comprising 269 patients were included. The mean age was 32 years and 58% were female. At diagnosis, 48% of patients with available cartilage data demonstrated advanced degeneration (grade ≥ 3). No statistically significant differences in recurrence risk were observed between treatment strategies; arthroscopic synovectomy, open synovectomy, and total hip arthroplasty. However, comparisons were limited by heterogeneous patient selection and follow-up duration. Arthroplasty was associated with the highest complication rate (33%), primarily aseptic loosening (19%). In the institutional cohort, 21.7% experienced recurrence and 29.4% required conversion to THA. Recurrences and conversions occurred exclusively in D-TGCT. PROMIS-CAT pain interference scores remained elevated (mean T-score 63.4 ± 11.1).

**Conclusion:**

Hip TGCT shows substantial cartilage damage at diagnosis and persistent postoperative morbidity. While recurrence rates appear comparable between surgical strategies, arthroplasty carries a higher complication burden in this predominantly young population. Prospective multicenter studies with standardized outcome reporting are required to optimize treatment strategies.

## Highlights

• Hip tenosynovial giant cell tumors frequently present with advanced cartilage damage at diagnosis.
• Recurrence risk appears comparable across surgical strategies.
• Arthroplasty demonstrates the highest complication burden.
• Persistent postoperative pain interference remains common.
• Early MRI evaluation may reduce diagnostic delay in young patients with unexplained hip pain.

## Graphical abstract

**Figure:**
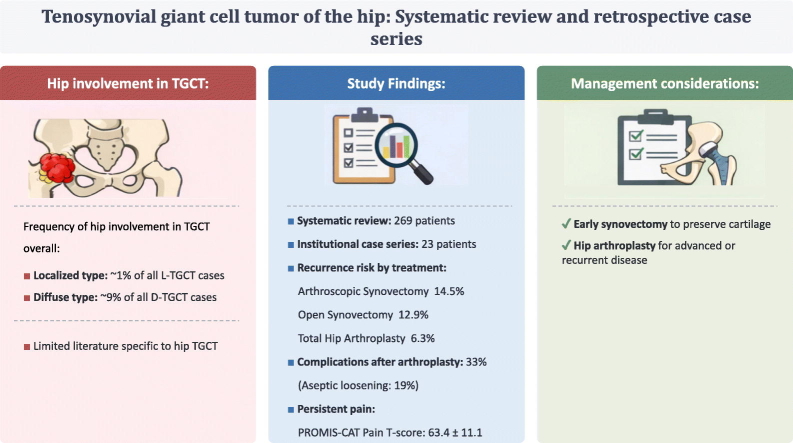

## 1 Introduction

**Fig. 1:**
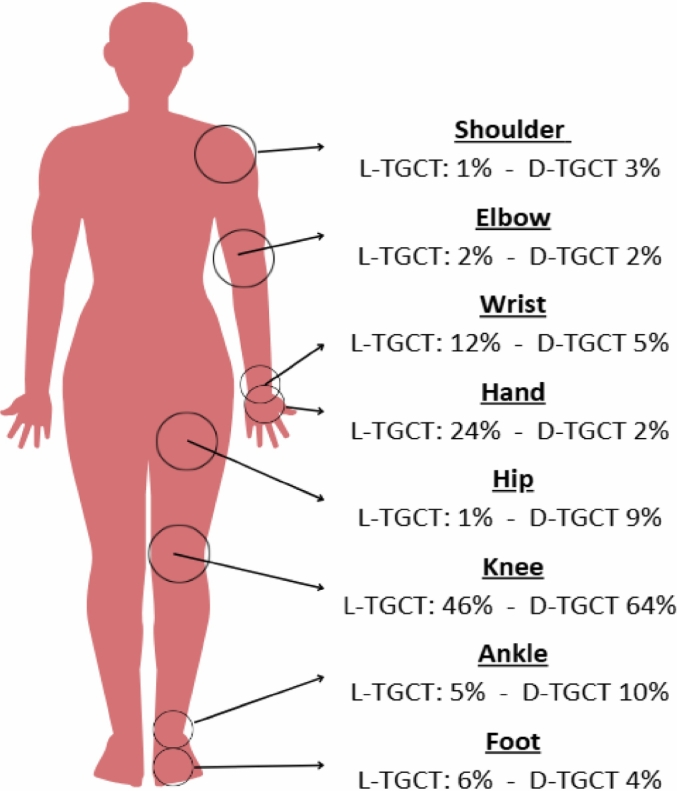
Anatomic distribution of TGCT in large joints.

Tenosynovial giant cell tumors (TGCT) are rare, predominantly benign but locally aggressive neoplasms arising from the synovium, bursae, and tendon sheaths, causing progressive joint destruction [1], [2]. Historically referred to as pigmented villonodular synovitis (PVNS), TGCT represents the current WHO terminology [3]. They are classified into localized (L-TGCT) and diffuse (D-TGCT) subtypes [3]. The knee is most commonly affected, whereas hip involvement is reported in approximately 1% of localized and 9% of diffuse cases (Fig. 1) [4].

The hip's deep anatomy and weight-bearing biomechanics may contribute to accelerated joint degeneration once synovial invasion occurs. The constrained synovial space may limit tumor expansion, potentially increasing intra-articular pressure and cartilage damage [5]. Diagnostic delay is common, as symptoms may mimic early osteoarthritis or soft tissue pathology [6], [7], [8]. Consequently, patients may present with advanced cartilage destruction at diagnosis [5].

Management typically consists of synovectomy, performed either arthroscopically or via an open approach [5], [9]. Total hip arthroplasty (THA) may be necessary in advanced disease. However, arthroplasty introduces implant-related complications in a relatively young patient population [5], [10]. The role of adjuvant radiotherapy and systemic CSF1R inhibitors remains evolving [11], [12], [13].

Data specific to hip TGCT remain limited and heterogeneous. Therefore, this study aimed to (1) systematically synthesize available literature on hip TGCT and (2) analyze two retrospective institutional cohorts with particular focus on recurrence risk and patient-reported outcomes.

## 2 Method

This study comprised a systematic review and a retrospective institutional case series. The systematic review followed PRISMA 2020 guidelines [14] and was prospectively registered in PROSPERO (CRD420251051854). Any protocol deviations were reported and justified.

Institutional data were retrieved from two tertiary referral centers. Ethical approval was obtained (METC 2025.0109).

### 2.1 Literature search

PubMed, Embase, and Cochrane Library were searched (January 1st 2000 – May 20th 2025) using search terms related to “tenosynovial giant cell tumor,” “TGCT,” “pigmented villonodular synovitis,” “PVNS,” and “hip.” (Appendix 1). Reference lists were screened manually. When studies potentially contained overlapping patient data, studies with larger cohorts and more comprehensive outcome measures were prioritized. This process was done independently by two researchers (EL & DM).

### 2.2 Eligibility criteria

Studies were eligible for inclusion if they reported original clinical data on patients diagnosed with hip TGCT, included ≥5 patients, reported recurrence, complications, functional outcomes, or patient-reported outcomes (PROMs). Reviews, editorials, conference abstracts, and studies before 2000 were excluded.

### 2.3 Study selection and critical appraisal

Two independent reviewers screened and extracted data. Discrepancies were resolved by a third reviewer (FV). Methodological quality was assessed using the MINORS criteria [15].

### 2.4 Meta-analysis

A random-effects model (DerSimonian–Laird method) was used due to anticipated heterogeneity [16]. Recurrence proportions were pooled using logit transformation. Statistical heterogeneity was assessed using the I^2^ statistic. Analyses were performed in R (version 4.5.1).

### 2.5 Case series

Patients with histologically confirmed hip TGCT were included. Patients lost to follow-up were described, and available data were included to minimize attrition bias. PROMIS-CAT questionnaires were administered [17]. For patients without PROMIS data, validated PROsetta Stone crosswalk conversion from SF-36 to PROMIS T-scores was applied [18].

## 3 Results

### 3.1 Systematic review

**Fig. 2:**
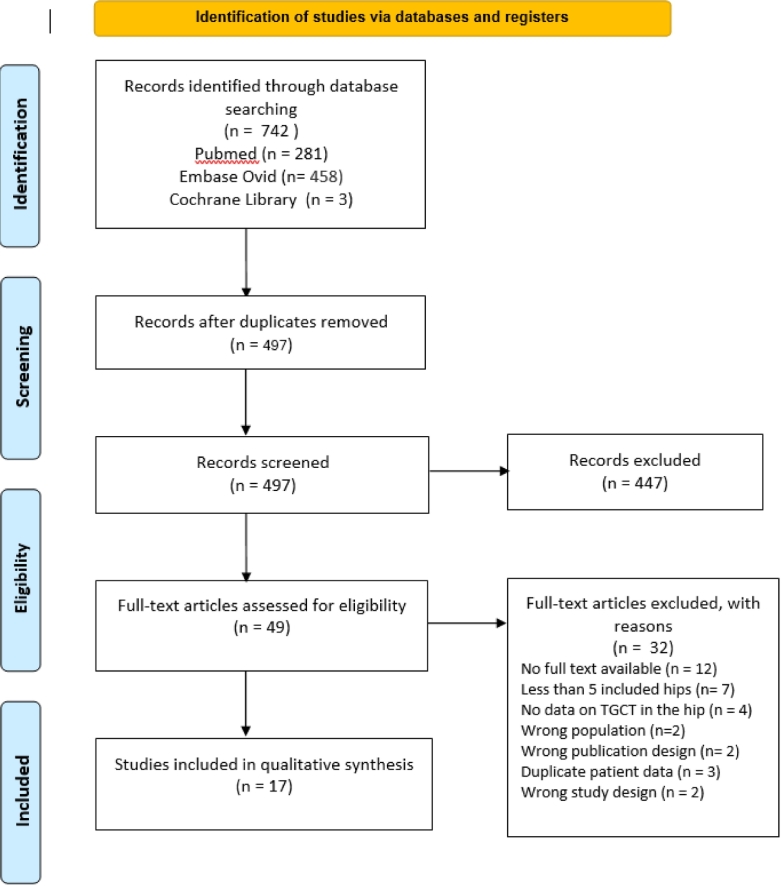
PRISMA flow diagram.

Seventeen studies comprising 269 patients were included (Table 2; Fig. 2) [8], [19], [20], [21], [22], [23], [24], [25], [26], [27], [28], [29], [30], [31], [32], [33], [34], [35]. Of the 17 included studies, 15 studies were non-comparative in design, and 2 were case-control studies. Geographical distribution included USA, Europe, and Asia, reflecting broad international representation.

The mean age was 32 years (range 7–68), and 58% were female (*n* = 145). Median reported diagnostic delay was 2.1 years (IQR 1.3–3.7), suggesting a prolonged symptomatic phase prior to definitive diagnosis in a substantial proportion of patients.

Subtype was reported in 8 studies: 58 patients (54%) had D-TGCT, 41 patients (38%) had L-TGCT, and in 9 patients (8%) the subtype was undefined [20], [23], [26], [27], [28], [29], [31], [34]. Follow-up duration ranged from 2 to 17 years (Table 2), although reporting was inconsistent and often lacked clear specification of median versus mean follow-up.

Among 141 patients with available cartilage data, 42 (30%) and 25 (18%) presented with grade 3 and grade 4 cartilage damage, respectively, indicating that 48% of evaluable patients already demonstrated advanced structural joint degeneration at diagnosis.

### 3.2 Critical appraisal

Quality assessment using the MINORS criteria indicated that 14 studies (82%) were of moderate quality and 3 studies (18%) were considered of poor quality (Table 1). No studies achieved high methodological quality (MINORS ≥15). Common methodological limitations included: (1) Lack of prospective enrollment (13 studies, 76%); (2) Inadequate specification of recurrence definitions (10 studies, 59%); (3) Absence of blinded outcome assessment (all studies); (4) Incomplete reporting of losses to follow-up (12 studies, 71%); (5) Failure to control for potential confounding variables (16 studies, 94%). These limitations introduce potential selection, detection, and reporting bias, which should be considered when interpreting pooled recurrence and complication rates. Detailed quality scores are provided in Appendix Table 1.

**Table 1:** Study characteristics included studies.

Author (year)	County	Study design (LoE)	No. Of hips	Therapy (No. of patients)	Adjuvant therapy (No. of patients)	Mean Follow-up years (range)
Byrd et al. (2013)	USA	Retrospective case series (IV)	13	Arthroscopic synovectomy	NR	5.25 (2−10)
Elzohairy et al. (2018)	Egypt	Retrospective case series (IV)	11	THA	No	7.2 (5–10.5)
Hufeland et al. (2017)	Germany	Retrospective case series (IV)	6	Arthroscopic synovectomy Open synovectomy	Radiotherapy for D-TGCT	8 (2.9–11.8)
Li et al. (2023)	China	Retrospective case series (IV)	37	Arthroscopic synovectomy THA	NR	4.2 ± 1.8 (2–7.8)
Ma et al. (2013)	China	Retrospective case series (IV)	12	Open synovectomy THA	No	NR
Nazal et al. (2020)	USA	Retrospective case series (IV)	14	Arthroscopic synovectomy	NR	6.66 ± 1.87
Ota et al. (2021)	Japan	Retrospective case series (IV)	10	Open synovectomy THA	No	5.5 (12–226)
Sun et al. (2022)	China	Retrospective case series (IV)	16	Arthroscopic synovectomy	Yes [8], No [8]	3.7 ± 3.2 (0.3–9.2)
Schenk et al. (2023)	Switzerland	Case control study (III)	18	Arthroscopic synovectomy Open synovectomy THA Conservative	Radiotherapy *n* = 2	5.4 ± 2.9
Tang et al. (2021)	Taiwan, Egypt, Germany	Retrospective case series (IV)	9	Arthroscopic synovectomy	Radiotherapy *n* = 5	4.7 (2–7)
Tibbo et al. (2018)	USA	Retrospective case series (IV)	25	THA	No	10
Willimon et al. (2018)	USA	Retrospective case series (IV)	5	Arthroscopic synovectomy	No	2.7 (1–5.3)
Xie et al. (2015)	China	Retrospective case series (IV)	43	Arthroscopic synovectomy Open synovectomy Synovectomy + THA	NR	NR
Xu et al. (2018)	Taiwain, China	Case control study (III)	19	THA	NR	8.6 (6.9–10.8)
Yoo et al. (2010)	South korea	Retrospective case series (IV)	8	THA	NR	8.9 (4.3–13.5)
Della valle et al. (2001)	Argentina	Retrospective case series (IV)	7	Open synovectomy THA Conservative	No	minimum of 2 years
Vastel et al. 2005)	France	Retrospective case series (IV)	16	Open synovectomy THA	no	16.7 (1−20)

LoE = level of evidence, NR = not reported, No. = number. THA = Total Hip Arthroplasty

**Table 2:** Patient characteristics.

Author (year)	No. of hips	Male/female	Age mean ± SD (range)	Subtype (L-TGCT/D-TGCT/undefined)	Diagnostic delay (years, range)	History of Trauma N (%)	Cartelige injury at diagnosis (grade) N (%)
Byrd et al. (2013)	13	9/4	26.8 (14–16)	3/3/7	1.4 (0.2–5.0)	NR	Grade 3: 3 (23) Grade 4: 4 (30)
Elzohairy et al. (2018)	11	6/5	38.2 (30–50)	NR	NR	NR	Grade 3: 11 (100)
Hufeland et al. (2017)	6	1/6	20.5 (14–27)	4/2	1.1 ± 0.72 (0.2–2.3)	NR	NR
Li et al. (2023)	37	16/22	36.35 ± 11.90 (21–66)	NR	NR	NR	Grade 4: 6 (17)
Ma et al. (2013)	12	NR	NR	NR	NR	NR	NR
Nazal et al. (2020)	14	6/8	32.69 ± 12.73	5/9	1,8 ± 2,43	NR	Grade 1: 8 (57) Grade 2: 4 (29) Grade 3: 1 (7)
Ota et al. (2021)	10	NR	NR	NR	NR	NR	*N* = 9 (90) no grade reported
Sun et al. (2022)	16	8/8	28.25 (18–66)	12/4	1.1 ± 1.40 (0.1–5.0)	NR	Grade 1: 2 (12.5) Grade 2: 4 (25) Grade 3: 4 (25) Grade 4: 6 (37.5)
Schenk et al. (2023)	18	8/10	35 (17–52)	3/15	NR	NR	NR
Tang et al. (2021)	9	2/7	24.3 ± 11.2 (14–44)	5/4	2.1 ± 0.2 (0.0–5.0)	NR	0 (0)
Tibbo et al. (2018)	25	9/16	39 (16–67)	25/0	2.9 ± 1.38 (1,0 - 6,2)	NR	NR
Willimon et al. (2018)	5	2/3	11 (7–17)	1/4	0.7 (0–2,6)	2 (40%)	NR
Xie et al. (2015)	43	17/26	32.07 ± 10.12	NR	NR	6 (13%)	NR
Xu et al. (2018)	19	8/14	35.2 (22–58)	NR	2.9 (1–6,2)	4 (25%)	NR
Yoo et al. (2010)	8	4/4	34.8 (20–68)	NR	8.3 (1–37)	NR	Grade 3: 3 (37.5) Grade 4: 5 (62)
Della valle et al. (2001)	7	2/5	27 (23−32)	NR	4.5 (0,7–11)	0 (0%)	Grade 3: 7 (100)
Vastel et al. 2005)	16	9/7	35.5 (23–61)	NR	5.7 ± 2.53 (1−10)	NR	Grade 2: 3 (18.8) Grade 3: 4 (25) Grade 4: 4 (25)

### 3.3 Patient-reported outcome measures (PROMs)

A broad range of PROMs was reported across the included studies. The most frequently utilized instruments were the (Modified) Harris Hip Score (mHHS/HHS) and the Visual Analogue Scale (VAS) for pain (Appendix Tables 2–4).

However, outcome reporting was heterogeneous with respect to timing, scoring methods, and baseline comparability, precluding formal pooling or subgroup analysis according to treatment strategy. Furthermore, only a minority of studies reported preoperative PROMs, limiting assessment of treatment-related improvement.

### 3.4 Treatment strategies

Three principal surgical strategies were identified: arthroscopic synovectomy, open synovectomy, and total hip arthroplasty (THA) (Appendix Table 5–8). Conservative management was infrequently reported and involved small numbers, precluding meaningful analysis.

**Fig. 3:**
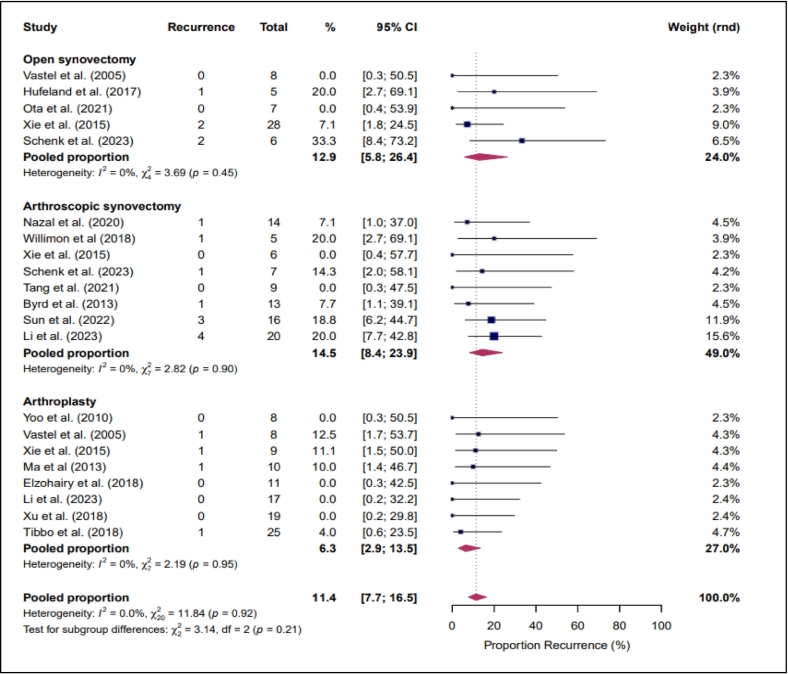
Meta-analyses on recurrence risk.

A meta-analysis comparing recurrence risk demonstrated no statistically significant differences between arthroscopic synovectomy, open synovectomy and arthroplasty (Fig. 3). Statistical heterogeneity across treatment subgroups was low (I^2^ = 0%, χ^2^ = 11.84, *p* = 0.92). The test for subgroup differences was not statistically significant (χ^2^ = 3.14, df = 2, *p* = 0.21) (Fig. 3).

Despite the absence of statistical heterogeneity, clinical heterogeneity in patient selection, disease stage, and follow-up duration must be acknowledged.

### 3.5 Arthroscopic and open synovectomy

Arthroscopic synovectomy included 91 patients across 9 studies (17, 23, 26, 45, 47, 50–52, 55). Open synovectomy included 57 patients across 7 studies [8], [19], [20], [22], [25], [26], [30].

Median follow-up was longer for open synovectomy (96.3 months (IQR: 60.2–141.9 months) compared with arthroscopic procedures (55.8 months (IQR: 47.6–68.0 months). This marked discrepancy in follow-up duration substantially limits direct comparison of recurrence rates between these surgical approaches and may underestimate recurrence in the arthroscopic group. Adjuvant radiotherapy was reported in 18 out of 91 patients (20%) undergoing arthroscopic synovectomy, and was not reported in the open synovectomy group.

Meta-analysis demonstrated a recurrence risk of 14.5% (95% CI 8.4–23.9) following arthroscopic synovectomy and 12.9% (95% CI 3.3–21.0) following open synovectomy.

Mean time to recurrence was reported in three studies after arthroscopic synovectomy and in two studies after open synovectomy. This resulted in a weighted mean time to recurrence of 59.3 months (range 44–77 months) and 64.5 months (range 21–108 months) respectively, suggesting comparable temporal recurrence patterns when recurrence occurred.

Conversion to arthroplasty after arthroscopic synovectomy was reported in 8 of 80 patients (10%) of whom this outcome was reported, after a median of 6 years (IQR: 4.8–6.2 years), all due to recurrent disease. After open synovectomy, 7 of 27 patients (26%) of whom this outcome was reported required conversion to arthroplasty after a median of 9 years (IQR: 5.2–10.3 years). However, reasons for conversion were inconsistently reported.

No procedure-related complications were reported in either treatment group, although underreporting cannot be excluded given the retrospective nature of most studies.

### 3.6 Arthroplasty

Arthroplasty was reported in 11 studies [8], [19], [21], [22], [25], [26], [29], [30], [33], [35], [36] including 115 patients. Four studies [28], [29], [33], [36] reported prior treatment before arthroplasty, but treatment details were inconsistently described.

Meta-analysis demonstrated the lowest pooled recurrence risk of 6.3% (95% CI 2.9–13.5) (Fig. 3). Four recurrences were reported; in two cases, the interval between arthroplasty and recurrence was specified as 14 and 24 years, indicating that very late recurrence remains possible even after joint replacement. Complications were reported in 38 of 114 patients (33.3%), most commonly aseptic loosening in 22 out of 114 patients (19%). Beyond aseptic loosening, a systematic extraction of complication data was performed across all included studies. This revealed that the majority of studies did not report individual complication types in sufficient detail to allow reliable quantification of specific adverse events such as dislocation or infection.

### 3.7 Institutional case series

A total of 25 patients were identified. Two patients were lost to follow-up, one continued follow-up elsewhere, and one declined further treatment, leaving 23 patients for analysis (Table 3). Of these, 16 patients (69.6%) had D-TGCT and 7 patients (30.4%) had L-TGCT (Appendix: Table 9).

**Table 3:** Summary of patient characteristics and treatment in case series.

Total included patients	23
Gender n (%)	
Female	17 (74)
Male	6 (26)
Mean age years (range)	32 (11–49)
Mean follow-up years (range)	6.2 (0.1–21.3)
TGCT subtype n (%)	
D-TGCT	16 (70)
L-TGCT	7 (30)
NR	1 ( [4]
Treatment n (%)	
Arthroscopic synovectomy	2 (8.7)
Open synovectomy	13 (56.5)
THA	5 (21.7)
Conservative	3 (13.0)
Adjuvant therapy	Cryotherapy n = 2 Radiotherapy n = 2
Recurrences n (%)	
Patients with ≥1 recurrence	5 (21.7)
Patients with ≥2 recurrences	1 (4.3)
Mean time to recurrence years (range)	1.9 ± 1.9 (0.5–5.3)
Conversion to arthroplasty n (%)	5 (29.4)
Mean time to conversion to Arthroplasty years (range)	1.8 ± 1.43 (0.5–3.8)

D-TGCT = Diffuse Tenosynovial Giant Cell tumor, L-TGCT = localized Tenosynovial Giant Cell tumor, THA = total hip arthroplasty

The cohorts had a female predominance (74%) with a median age of 32 (IQR: 22–41) years. Median follow-up was 4.6 years (IQR: 1.2–9.8 years).

Open synovectomy was most frequently performed (13 patients, 56.7%), followed by THA (5, 21.7%) and arthroscopic synovectomy (2, 8.7%). Conservative management was chosen in 3 patients (13%).

Adjuvant treatments included radiotherapy (*n* = 2), and cryotherapy (*n* = 1).

Four patients had one recurrence and one patient had two recurrences, resulting in a recurrence rate of 21.7%. All recurrences occurred exclusively in patients with D-TGCT while no recurrences were observed in the L-TGCT subgroup. Recurrences were treated with open synovectomy (*n* = 1), arthroscopic synovectomy (n = 1), cup revision (n = 1), or arthroplasty (*n* = 3). Adjuvant radiotherapy was used in three cases.

Five out of 18 patients (27%) not initially treated with arthroplasty required conversion to THA. All conversions occurred in patients with D-TGCT. The median time to conversion was 1.5 years (interquartile range (IQR), 2.6 years; range 0.5–22). Reasons for conversion were persistent pain (*n* = 2), recurrence (n = 2) and avascular necrosis (*n* = 1). Notably, the relatively short median time to conversion suggests that progression to arthroplasty may occur early in selected patients despite initial joint-preserving surgery.

Complications included avascular necrosis (n = 1) after arthroscopic synovectomy, requiring conversion to THA and direct post-operative dislocation (n = 1) after THA. Two patients required removal of trochanteric fixation screws because of persistent lateral hip pain following open synovectomy with concomitant trochanteric osteotomy.

### 3.8 PROMIS-CAT outcomes

The case series revealed elevated PROMIS-CAT pain interference scores (mean T-score 63.4 ± 11.1), indicating pain levels substantially above population norms despite surgical intervention. PROMIS T-scores are standardized to a general population mean of 50 (SD = 10), such that a pain interference score of 63.4 represents more than one standard deviation above the population norm, indicating clinically meaningful residual pain burden in this cohort. Scores above 60 are generally considered to reflect moderate-to-severe symptom burden in clinical research contexts. These findings suggest that disease- and treatment-related morbidity may persist despite oncologic control. Other PROMIS-CAT domains approached population averages (Appendix: Table 10).

## 4 Discussion

To our knowledge, this study provides the most comprehensive synthesis to date of tenosynovial giant cell tumor (TGCT) of the hip, combining a systematic review (269 patients) with two institutional case series (23 patients). The present findings indicate that hip TGCT follows a more aggressive clinical course than TGCT in other joints, characterized by delayed diagnosis, advanced cartilage damage at presentation, and persistent postoperative morbidity despite treatment.

Hip TGCT appears to present with higher rates of advanced cartilage damage compared to knee TGCT in available literature (48% vs. 18%) [25], reflecting both underlying disease biology and diagnostic delays in this location [5]. However, direct comparative studies are lacking. The deep anatomical location of the hip joint, combined with its constrained synovial volume, may promote early intra-articular pressure increase and accelerate cartilage degradation once synovial proliferation occurs [5]. In addition, weight-bearing biomechanics likely further exacerbate mechanical cartilage wear [5]. At the same time, the deep location of the joint may obscure early clinical symptoms, contributing to a median diagnostic delay of 2.1 years [5] [6]. Importantly, this delay appears to have direct clinical consequences, as nearly half of patients already demonstrate advanced cartilage damage at the time of diagnosis, suggesting a missed window for joint-preserving intervention [6], [7]. The relationship between osseous erosion and local recurrence risk has recently been described in knee TGCT, where Olson et al. demonstrated that all patients with D-TGCT and osseous erosions developed local recurrence, compared to 37% without erosions (*p* = 0.037) [37]. Whether osseous erosion carries similar prognostic implications in the hip remains unknown, as none of the included studies systematically analyzed the relationship between cartilage damage grade and recurrence risk in this location, representing an important gap for future research.

These findings suggest that hip TGCT should not be extrapolated from knee-based evidence, but considered a distinct clinical entity requiring tailored management strategies. This has important implications for clinical practice, as early recognition and timely imaging may represent the only opportunity to preserve joint integrity in this predominantly young patient population.

An important finding in the case series was the persistently elevated PROMIS-CAT pain interference score (T-score 63.4), indicating substantial residual symptom burden despite surgical treatment [17]. This observation underscores that disease impact extends beyond oncologic control or implant survival, highlighting the importance of functional and patient-reported outcomes in evaluating treatment success. In contrast, the systematic review demonstrated highly heterogeneous reporting of PROMs, with variable use of instruments such as the Harris Hip Score, WOMAC, and VAS, precluding pooled analysis. The use of PROMIS-CAT in the present study represents a strength, as it provides a standardized and validated assessment of patient-centered outcomes, allowing for more meaningful interpretation of long-term functional burden [17].

Adjuvant radiotherapy was rarely reported in the included studies, resulting in insufficient data to establish efficacy. Systemic CSF1R inhibitors may represent an alternative for unresectable or recurrent D-TGCT [38], [39], although long-term functional benefit and durability remain unclear [13]. Future studies should evaluate whether combined treatment strategies, including limited surgery and systemic therapy, may delay or prevent the need for arthroplasty in young patients with diffuse disease.

The findings of this study should be interpreted in the context of several limitations. First, the overall methodological quality of included studies was moderate to low, with the majority of studies being retrospective and lacking standardized outcome reporting. Second, substantial heterogeneity in patient selection, treatment strategies, follow-up duration, and postoperative surveillance protocols limits robust direct comparison between treatment modalities. Third, definitions of recurrence and complications were inconsistently reported, and few studies distinguished clearly between persistent and recurrent disease. Fourth, complication reporting after arthroplasty was highly heterogeneous across included studies; beyond aseptic loosening, individual adverse events such as dislocation and infection were inconsistently documented, precluding reliable extraction of absolute numbers for specific complication types. Finally, the absence of standardized reporting of cartilage damage and PROMs across studies further limits the strength of pooled analyses.

Notably, only two studies reported outcomes stratified by TGCT subtype, precluding meaningful subgroup analyses. This represents an important gap in the literature, as biological behavior and treatment response may differ substantially between localized and diffuse disease. Future research should focus on standardized definitions, uniform reporting of cartilage damage, and prospective multicenter data collection. In this context, the development of national or international registries may facilitate robust evaluation of disease course, treatment strategies, and long-term outcomes in this rare condition.

The meta-analysis demonstrated no statistically significant differences in recurrence risk between arthroscopic synovectomy, open synovectomy, and THA. However, direct comparison between synovectomy and THA is inherently limited, as THA eliminates the native joint and substantially reduces residual synovial tissue, likely contributing to the lower pooled recurrence risk in this group. Nevertheless, as TGCT originates from synovial tissue that may extend beyond the joint capsule, recurrence after arthroplasty remains possible, as evidenced by cases occurring 14 and 24 years postoperatively in this review. Additionally, the discrepancy in follow-up duration between arthroscopic and open synovectomy (55.8 vs. 96.3 months) likely underestimates true recurrence in the arthroscopic group. It should further be noted that none of the included studies described a standardized surveillance protocol. Asymptomatic patients are unlikely to return to the treating center after routine follow-up ends, whereas patients developing recurrence typically experience a return of symptoms prompting renewed clinical contact, likely distorting both recurrence rates and time-to-recurrence estimates across all treatment groups. Finally, with treatment subgroups ranging from 57 to 115 patients and low baseline recurrence rates (6–15%), statistical power was insufficient to reliably detect clinically meaningful differences between modalities, and a type II error cannot be excluded.

From a clinical perspective, the present findings suggest synovectomy as the preferred initial treatment in patients with preserved cartilage, while arthroplasty should be reserved as a salvage procedure for advanced or recurrent disease. However, given the absence of comparative study designs in the available literature, no formal treatment hierarchy can be established. The substantial complication rates observed after arthroplasty, particularly in a relatively young patient population, warrant careful patient selection and thorough preoperative counseling. The role of CSF1R inhibitors such as pexidartinib in hip TGCT remains unexplored in the included studies. Although systemic CSF1R inhibition has shown efficacy in diffuse TGCT at other anatomical sites, its specific value in hip disease, whether as primary therapy, neoadjuvant treatment, or an alternative to arthroplasty in young patients with diffuse disease, warrants prospective evaluation [40].

Furthermore, clinicians should maintain a high index of suspicion for TGCT in young adults presenting with unexplained hip pain or imaging findings suggestive of early osteoarthritis [6], [7]. Early MRI should be considered to reduce diagnostic delay [6], [8]. A multidisciplinary approach involving orthopaedic oncologists, radiologists, and rehabilitation specialists is likely essential to optimize both oncologic and functional outcomes.

## 5 Conclusion

TGCT of the hip is a rare but clinically impactful disease associated with significant morbidity, long diagnostic delays, and high rates of cartilage damage at presentation. The hip's unique anatomical and biomechanical characteristics explain its distinctly different natural history compared to TGCT in other joints. Initial synovectomy is appropriate for patients with relatively preserved cartilage, while arthroplasty should be reserved for advanced or recurrent disease. Standardized reporting, prospective multicenter collaboration, and long-term PROM evaluation are essential to optimize evidence-based treatment algorithms.

## Declaration of generative AI and AI-assisted technologies in the manuscript preparation process

During the preparation of this work the author(s) used Perplexity AI (version 2025) in order to improve the readability, language quality, and organization of the manuscript. After using this tool/service, the author(s) reviewed and edited the content as needed and take full responsibility for the content of the published article.

## CRediT authorship contribution statement

**E. van der Loos:** Writing – review & editing, Writing – original draft, Visualization, Project administration, Methodology, Investigation, Formal analysis, Data curation, Conceptualization. **D.T. Meijer:** Writing – review & editing, Validation, Supervision. **J.A.M. Bramer:** Supervision. **H.W.B. Schreuder:** Writing – review & editing, Supervision. **F.G.M. Verspoor:** Writing – review & editing, Visualization, Validation, Supervision, Investigation, Data curation, Conceptualization.

## Funding

This research did not receive any specific grant from funding agencies in the public, commercial, or not-for-profit sectors.

## Declaration of competing interest

The authors declare no competing interests.

The authors declare that they have no known competing financial interests or personal relationships that could have appeared to influence the work reported in this paper.

Graphical abstract.
