# Supplementary material for: Tenosynovial giant cell tumor of the hip: a systematic review and institutional case series with Meta-analysis of recurrence and patient-reported outcomes
Source: J Bone Oncol. 2026 May 25;58:100769. doi: 10.1016/j.jbo.2026.100769 (PMC13241937; doi:10.1016/j.jbo.2026.100769)
Supplement: Supplementary file 1 — Supplementary material 1 [file mmc1.docx]

## Appendix 1: Full search strategy

**PUBMED**

("Giant Cell Tumor of Tendon Sheath"[Mesh] OR "Synovitis, Pigmented Villonodular"[Mesh] OR tenosynovial giant cell tumo*[tiab] OR TGCT[tiab] OR "Giant Cell Tumor of the Tendon Sheath"[tiab] OR GCTTS[tiab] OR pigmented villonodular synovitis[tiab] OR PVNS[tiab] OR pigmented villonodular tenosynovitis[tiab] OR localized nodular synovitis[tiab] OR villonodular synovitis[tiab] OR hemosiderotic synovitis[tiab] OR synovial giant cell tumo*[tiab])

**AND**

("Hip"[Mesh] OR  "Hip Joint"[Mesh] OR hip[tiab] OR hips[tiab] OR coxal[tiab] OR coxae[tiab] OR coxofemoral[tiab] OR femoroacetabular[tiab])

**EMBASE OVID:**

Database(s): Embase Classic+Embase up to 20^th^ of May 2025
Search Strategy:

| # | Searches |  |
| --- | --- | --- |
| 1 | "giant cell tumor of tendon sheath"/ or pigmented villonodular synovitis/ |  |
| 2 | (tenosynovial giant cell tumo* or TGCT or "Giant Cell Tumor of the Tendon Sheath" or GCTTS or pigmented villonodular synovitis or PVNS or pigmented villonodular tenosynovitis or localized nodular synovitis or villonodular synovitis or hemosiderotic synovitis or synovial giant cell tumo*).ti,ab,kf. |  |
| 3 | 1 or 2 |  |
| 4 | exp hip/ or exp hip joint/ |  |
| 5 | (hip or hips or coxal or coxae or coxofemoral or femoroacetabular).ti,ab,kf. |  |
| 6 | 4 or 5 |  |
| 7 | 3 and 6 |  |

**Cochrane Library**

#1 (tenosynovial giant cell tumo* or TGCT or "Giant Cell Tumor of the Tendon Sheath" or GCTTS or pigmented villonodular synovitis or PVNS or pigmented villonodular tenosynovitis or localized nodular synovitis or villonodular synovitis or hemosiderotic synovitis or synovial giant cell tumo*):ti,ab,kw

#2 (hip or hips or coxal or coxae or coxofemoral or femoroacetabular):ti,ab,kw

#3 #1 and #2
