# Supplementary material for: Tenosynovial giant cell tumor of the hip: a systematic review and institutional case series with Meta-analysis of recurrence and patient-reported outcomes
Source: J Bone Oncol. 2026 May 25;58:100769. doi: 10.1016/j.jbo.2026.100769 (PMC13241937; doi:10.1016/j.jbo.2026.100769)
Supplement: Supplementary file 2 — Supplementary material 2 [file mmc2.docx]

## Table 1: Critical appraisal included studies using the MINORS criteria

|  | 1. A clearly stated aim | 2. Inclusion of consecutive patients | 3. Prospective collection of data | 4. Endpoints appropriate to the aim of the study | 5. Unbiased assessment of the study endpoint | 6. Follow-up period appropriate to the aim of the study | 7. Loss to follow up less than 5% | 8. Prospective calculation of the study size | 9. An adequate control group | 10. Contemporary groups | 11. Baseline equivalence of groups: | 12. Adequate statistical analyses | **Total score** | Overall assessment |
| --- | --- | --- | --- | --- | --- | --- | --- | --- | --- | --- | --- | --- | --- | --- |
| **Byrd et al. (2013)** | 2 | 2 | 0 | 2 | 2 | 2 | 2 | 0 | NA | NA | NA | NA | 12 | M |
| **Elzohairy et al. (2018)** | 2 | 2 | 0 | 2 | 2 | 2 | 2 | 0 | NA | NA | NA | NA | 12 | M |
| **Hufeland et al. (2017)** | 2 | 2 | 0 | 2 | 2 | 2 | 2 | 0 | NA | NA | NA | NA | 12 | M |
| **Li et al. (2023)** | 2 | 2 | 0 | 2 | 2 | 1 | 2 | 0 | NA | NA | NA | NA | 11 | M |
| **Ma et al (2013)** | 2 | 2 | 0 | 2 | 1 | 1 | 0 | 0 | NA | NA | NA | NA | 8 | P |
| **Nazal et al. (2020)** | 2 | 2 | 0 | 2 | 2 | 2 | 2 | 0 | NA | NA | NA | NA | 12 | M |
| **Nishida et al. (2012)** | 2 | 2 | 0 | 2 | 2 | 2 | 2 | 0 | NA | NA | NA | NA | 12 | M |
| **Ota et al. (2021)** | 2 | 2 | 0 | 2 | 2 | 2 | 1 | 0 | NA | NA | NA | NA | 11 | M |
| **Sun et al. (2022)** | 2 | 2 | 0 | 2 | 0 | 1 | 1 | 0 | NA | NA | NA | NA | 8 | P |
| **Schenk et al. (2023)** | 2 | 2 | 0 | 2 | 1 | 2 | 1 | 0 | 0 | 2 | 0 | 2 | 14 | P |
| **Tang et al. (2021)** | 2 | 2 | 0 | 2 | 1 | 1 | 1 | 0 | NA | NA | NA | NA | 9 | M |
| **Tibbo et al. (2018)** | 2 | 2 | 0 | 2 | 2 | 2 | 2 | 0 | NA | NA | NA | NA | 12 | M |
| **Willimon et al (2018)** | 2 | 2 | 0 | 2 | 1 | 1 | 2 | 0 | NA | NA | NA | NA | 10 | M |
| **Xie et al. (2015)** | 2 | 2 | 0 | 2 | 1 | 0 | 0 | 0 | NA | NA | NA | NA | 7 | P |
| **Xu et al. (2018)** | 2 | 2 | 0 | 2 | 1 | 2 | 1 | 0 | 2 | 2 | 2 | 2 | 18 | M |
| **Yoo et al. (2010)** | 2 | 2 | 0 | 2 | 1 | 2 | 2 | 0 | NA | NA | NA | NA | 11 | M |
| **Della valle et al. (2001)** | 2 | 1 | 0 | 2 | 1 | 1 | 2 | 0 | NA | NA | NA | NA | 9 | M |
| **Vastel et al. (2005)** | 2 | 2 | 0 | 2 | 1 | 2 | 2 | 0 | NA | NA | NA | NA | 11 | M |
| *2 = adequately scored, 1 = inadequately reported, 0 = not reported or not applicable. NA = Not Applicable*  *P = Poor quality, M = Moderate quality* | | | | | | | | | | | | | | |
