# Supplementary material for: Tenosynovial giant cell tumor of the hip: a systematic review and institutional case series with Meta-analysis of recurrence and patient-reported outcomes
Source: J Bone Oncol. 2026 May 25;58:100769. doi: 10.1016/j.jbo.2026.100769 (PMC13241937; doi:10.1016/j.jbo.2026.100769)
Supplement: Supplementary file 3 — Supplementary material 3 [file mmc3.docx]

## Table 2: Patient reported outcome measures after arthroscopic synovectomy

| Author (year) | Used PROMs | HHS & mHHS  MEAN (sd) | VAS  mean (sd) | Other  Mean (sd) |
| --- | --- | --- | --- | --- |
| Byrd et al. (2013) | HHS | Improvement: 89 (12)  Initial: 27 (17)  Follow-up: 62 (14) | - | - |
| Hufeland et al. (2017) | mHHS | Follow-up: 91.04 (12.3) | - | - |
| Li et al. (2023) | HHS, VAS | Improvement: 26.30 (21.38)  Initial: 45.30 (11.08)  Follow-up: 71.60 (19.78) | Improvement: 1.70 (2.02)  Initial: 4.9 (1.7)  Follow-up: 1.35 (1.79) | - |
| Nazal et al. (2020) | mHHS, VAS | Initial: 74.08 (16.84) | Improvement: 4.9 (3.7)  Initial: 8.1 (1.1)  Follow-up: 3.2 (1.8) | *iHot-12*  Follow-up: 67.9 (27.4)  *HOS-ADL*  Follow-up: 57.5 (12.5) |
| Sun et al. (2022) | mHHS, HHS, VAS | Improvement: 12.8  Initial: 63.2 (8.1)  Follow-up: 75.9 (12.5) | Improvement: 1.8  Initial: 6.0 (1.6)  Follow-up: 3.2 (1.6) | *iHot-12*  Improvement: 27.9  Initial: 45.1  Follow-up: 73.1 |
| Sun et al. (2022) | mHHS, HHS, VAS | Improvement: 29.0  Initial: 52.6 (25.4)  Follow-up: 81.6 (6.3) | Improvement: 2.2  Initial: 6.3 (1.1)  Follow-up: 3.1 (0.9) | *HOS-ADL*  Improvement: 20.3  Initial: 51.1  Follow-up: 81.4  *iHot-12*  Improvement: 19.4  Initial: 55.9  Follow-up: 75.3 |
| Schenk et al. (2023) | none | - | - | - |
| Tang et al. (2021) | mHHS | Follow-up: 94.6 (4.9) | - | *HOS-ADL*  Mean: 93.3 (20.2) |
| Willmon et al. (2018) | None | - | - | - |
| Xie et al. (2015) | None | - | - | - |

*NR = not reported, PROM = Patient Reported Outcome Measure, (m)HHS = (modified) Harris hip score, VAS = Visual Analogue Scale, iHOT = international Hip Outcome Tool -12, HOS-ADL = Hp Outcome Score – Activities of Daily Living subscale*
