# Supplementary material for: Tenosynovial giant cell tumor of the hip: a systematic review and institutional case series with Meta-analysis of recurrence and patient-reported outcomes
Source: J Bone Oncol. 2026 May 25;58:100769. doi: 10.1016/j.jbo.2026.100769 (PMC13241937; doi:10.1016/j.jbo.2026.100769)
Supplement: Supplementary file 4 — Supplementary material 4 [file mmc4.docx]

## Table 3: Patient reported outcome measures after open synovectomy

| Author (year) | Used pROM | mHHS improvement  (SD, range) | mHHS initial | mHHS follow-up |
| --- | --- | --- | --- | --- |
| Hufeland et al. (2017) | mHHS | 88.8 (14.82) (7.1 - 100) | NR | NR |
| Ma et al (2013) | None | - | - | - |
| Ota et al. (2021) | None | - | - | - |
| Schenk et al. (2023) | None | - | - | - |
| Xie et al. (2015) | None | - | - | - |
| Della valle et al. (2001) | None | - | - | - |
| Vastel et al. (2005) | None | - | - | - |

MHHS = modified Harris Hip Score
