# Supplementary material for: Tenosynovial giant cell tumor of the hip: a systematic review and institutional case series with Meta-analysis of recurrence and patient-reported outcomes
Source: J Bone Oncol. 2026 May 25;58:100769. doi: 10.1016/j.jbo.2026.100769 (PMC13241937; doi:10.1016/j.jbo.2026.100769)
Supplement: Supplementary file 5 — Supplementary material 5 [file mmc5.docx]

## Table 4: Patient reported outcome measures after total hip arthroplasty

| Author (year) |  | Used PROM | (m)HHS mean (Sd) (Range) | VAS | Other  mean (Sd) (Range) |
| --- | --- | --- | --- | --- | --- |
| Elzohairy et al. (2018) |  | mHHS | Improvement: 48.2  Initial: 46.1 (30-70)  Follow-up: 94.3 (90 - 100) | - | - |
| Ma et al (2013) |  | None | - | - | - |
| Xie et al. (2015) |  | None | - | - | - |
| Yoo et al. (2010) |  | HHS | Improvement: 47.4 (11.7) (28-58)  Initial: 49.3 (14.1) (34 - 72)  Follow-up: 96.6 (3.4) (92.0 - 100) | - | - |
| Vastel et al. (2005) |  | None | - | - | - |
| Li et al. (2023) |  | HHS, VAS | Improvement: 33,96  Initial: 45.24 (10.36)  Follow-up: 78.94 (19.11) | Improvement: 3.30 (1.06)  Initial: 3.65 (0.79)  Improvement: 0.35 (0.70) |  |
| Ota et al. (2021) |  | None | - | - | - |
| Schenk et al. (2023) |  | iHOT-12 | - | - | *iHOT-12*  Follow-up: 93.3(20.2) (50–120) |
| Tibbo et al. (2018) |  | HHS | Improvement: 30  Initial: 48 (23-69)  Follow-up: 78 (47-96) | - | - |
| Xu et al. (2018) |  | HHS | Improvement: 43.9  Initial: 48.7 (3.8) (39 - 62)  Follow-up: 92.6 (5.5) (81-99) | - | - |
| Della valle et al. (2001) |  | None | - | - | - |

*NR = not reported, PROM = Patient Reported Outcome Measure, (m)HHS = (modified) Harris hip score, VAS = Visual Analogue Scale, iHOT = international Hip Outcome Tool -12*
