# Supplementary material for: Tenosynovial giant cell tumor of the hip: a systematic review and institutional case series with Meta-analysis of recurrence and patient-reported outcomes
Source: J Bone Oncol. 2026 May 25;58:100769. doi: 10.1016/j.jbo.2026.100769 (PMC13241937; doi:10.1016/j.jbo.2026.100769)
Supplement: Supplementary file 6 — Supplementary material 6 [file mmc6.docx]

## Appendix Table 5: Outcomes after arthroscopic synovectomy

| Author (year) | No. of patients | Adjuvant therapy  (radiotherapy)  (n) | Subtype  (L-TGCT /  D-TGCT / undefined) | Prior treatment | Recurrence No. | mean Time to recurrence (years) | Secondary THA No. | Time between first operation till THA (years) |
| --- | --- | --- | --- | --- | --- | --- | --- | --- |
| Byrd et al. (2013) | 13 | NR | 3/3/7 | NR | 1 | NR | 1 | 6 |
| Hufeland et al. (2017) | 1 | NR | 0/1 | NR | 0 | NR | 0 | NR |
| Li et al. (2023) | 20 | NR | - | NR | 4 | 3.7 ± 0.58 | 4 | 3.7 ± 0.58 |
| Nazal et al. (2020) | 14 | NR | 5/9 | NR | 1 | 5.4 | 0 | NR |
| Sun et al. (2022) | 16 | 8 | 6/2 | NR | 0 | 6.4 | 0 | 6.4 |
| Schenk et al. (2023) | 7 | NR | NR | NR | 1 | NR | 1 | NR |
| Tang et al. (2021) | 9 | 5 | 5/4 | N = 9 (undefined) | 0 | NR | 0 | NR |
| Willimon et al (2018) | 5 | No | 1/4 | no | 1 | NR | NR | NR |
| Xie et al. (2015) | 6 | NR | NR | NR | 1 | NR | NR | NR |
| *NR = not reported. No. = number. L-TGCT = localized tenosynovial giant cell tumor, D-TGCT = diffuse Tenosynovial giant cell tumor, THA = total hip arthroplasty* | | | | | | | | |
