# Supplementary material for: Tenosynovial giant cell tumor of the hip: a systematic review and institutional case series with Meta-analysis of recurrence and patient-reported outcomes
Source: J Bone Oncol. 2026 May 25;58:100769. doi: 10.1016/j.jbo.2026.100769 (PMC13241937; doi:10.1016/j.jbo.2026.100769)
Supplement: Supplementary file 7 — Supplementary material 7 [file mmc7.docx]

## Table 6: Outcomes after open synovectomy

| **Author (year)** | **No of patients** | **Therapy details** | **Adjuvant therapy** | **Subtype**  **(L-TGCT / D-TGCT )** | **Recurrence** | **Years to recurrence (mean)** | **Second recurrence** | **Secondairy osteoartritis progression** | **Secondary THA No.** | **years between first operation till THA** | **Revisions No.** |
| --- | --- | --- | --- | --- | --- | --- | --- | --- | --- | --- | --- |
| ***Hufeland et al. (2017)*** | 5 | NR | 1 | 4/1 | 1 | 1.8 | 1 | NR | 1 | 1,3 | 0 |
| ***Ma et al (2013)*** | 2 | NR | No | NR | 1 | NR | NR | NR | NR | NR | NR |
| ***Ota et al. (2021)*** | 7 | NR | NR | NR | 0 | NR | NR | 5 | 1 | NR | NR |
| ***Schenk et al. (2023)*** | 6 | Dislocation n= 4, Minimal invasive n=2 | NR | NR | 2 | NR | NR | NR | 0 | NR | NR |
| ***Xie et al. (2015)*** | 28 | NR | NR | NR | 2 | NR | NR | NR | NR | NR | NR |
| ***Della valle et al. (2001)*** | 1 | NR | No | NR | 1 | 9 | NR | NR | 1 | 9 | 0 |

*NR = not reported. No. = number. L-TGCT = localized tenosynovial giant cell tumor, D-TGCT = diffuse tenosynovial giant cell tumor, THA = total hip arthroplasty*
