# Supplementary material for: Tenosynovial giant cell tumor of the hip: a systematic review and institutional case series with Meta-analysis of recurrence and patient-reported outcomes
Source: J Bone Oncol. 2026 May 25;58:100769. doi: 10.1016/j.jbo.2026.100769 (PMC13241937; doi:10.1016/j.jbo.2026.100769)
Supplement: Supplementary file 8 — Supplementary material 8 [file mmc8.docx]

## Table 7: Outcomes after total hip arthroplasty

| Author (year) | No of patients | Therapy details | Adjuvant therapy | Subtype  (L-TGCT . D-TGCT ) | Prior treatment | Recurrence NO. | Time to recurrence (mean, months) | Complications | Revisions No |
| --- | --- | --- | --- | --- | --- | --- | --- | --- | --- |
| *Elzohairy et al. (2018)* | 11 | Cementless | No | NR | NR | 0 | NR | 0 | NR |
| *Ma et al (2013)* | 10 | NR | No | NR | NR | 1 | NR | NR |  |
| *Xie et al. (2015)* | 9 | NR | NR | NR | NR | 1 | NR | NR | NR |
| *Yoo et al. (2010)* | 8 | Cementless | NR | NR | Open synovectomy n = 3 | 0 | NR | Aseptic loosening n=2 | 2 |
| *Vastel et al. (2005)* | 8 | Cementing n = 4  Cup arthroplasty n=3 Monopolar n =1 | No | NR | NR | 1 | 14 years | Aseptic loosening n=2 | 1 |
| *Li et al. (2023)* | 17 | NR | NR | NR | NR | 0 | NR | Aseptic loosening n=3  Unspecified complications n=8 | 5 |
| *Ota et al. (2021)* | 3 | NR | NR | NR | NR | 0 | NR | NR | NR |
| *Schenk et al. (2023)* | 1 | NR | NR | NR | NR | 0 | NR | NR | NR |
| *Tibbo et al. (2018)* | 25 | Cemented n = 7  Uncemented = 13  Hybrid n = 3 Resurfacing n = 2 | No | 25/0 | 16 Unspecified | 1 | 24 years | Aseptic loosening n=12  Osteolyses secondary to polyethylen wear n = 1  Early postoperatieve deep infection n=1  Acetabular erosion n=1  Painful bipolar hemiarthroplasty. N=1 | 16 |
| *Xu et al. (2018)* | 19 | Cementlesss | NR | NR | Arthroscpoic synovectomy n =5 * | 0 | NR | Aseptic loosening n=1  Squaeking n=1 | 1 |
| *Della valle et al. (2001)* | 4 | NR | No | NR | NR | 0 | NR | Aseptic loosening n=2 | 2 |
| * mean years before THA: 2,4 range 0,8 NR 3,6  NR = Not reported, L-TGCT = localized tenosynovial giant cell tumor, D-TGCT = diffuse tenosynovial giant cell tumor | | | | | | | | | |
