# Supplementary material for: Tenosynovial giant cell tumor of the hip: a systematic review and institutional case series with Meta-analysis of recurrence and patient-reported outcomes
Source: J Bone Oncol. 2026 May 25;58:100769. doi: 10.1016/j.jbo.2026.100769 (PMC13241937; doi:10.1016/j.jbo.2026.100769)
Supplement: Supplementary file 9 — Supplementary material 9 [file mmc9.docx]

## Table 8: Outcomes after conservative treatment

| Author (year) | No. of patients | Adjuvant therapy | Subtype  (L-TGCT /  D-TGCT / undefined) | Progression No. | Time to recurrence (mean, months) | Secondairy osteoartritis progression | Secondary THA No. |
| --- | --- | --- | --- | --- | --- | --- | --- |
| *Schenk et al. (2023)* | 4 | No | 0/4 | 2 | NR | 0 | 0 |
| *Della valle et al. (2001)* | 2 | No | NR | 1 | NR | 1 | NR |

*NR = not reported. No. = number. L-TGCT = localized tenosynovial giant cell tumor, D-TGCT = diffuse tenosynovial giant cell tumor, THA = total hip arthroplasty.*
