# Supplementary material for: Tenosynovial giant cell tumor of the hip: a systematic review and institutional case series with Meta-analysis of recurrence and patient-reported outcomes
Source: J Bone Oncol. 2026 May 25;58:100769. doi: 10.1016/j.jbo.2026.100769 (PMC13241937; doi:10.1016/j.jbo.2026.100769)
Supplement: Supplementary file 10 — Supplementary material 10 [file mmc10.docx]

## Table 9: Overview of included patients in the case series

| Database | ID | Gender (m/f) | Age at diagnosis | Subtype (diffuse/ localized) | Follow-up (years) | Initial treatment | Adjuvant treatment | Complications yes/no | No. Recurrences | Time until first recurrence (years) | Recurence treatment | Conversion to THA | Duration until THA (years) |
| --- | --- | --- | --- | --- | --- | --- | --- | --- | --- | --- | --- | --- | --- |
| Amsterdam UMC | 1 | F | 23 | D-TGCT | 1.1 | Conservative | No | No | 0 | N/A | N/A | - | N/A |
|  | 2 | F | 20 | L-TGCT | 1.3 | Open synovectomy | No | No | 0 | N/A | N/A | - | N/A |
|  | 3 | F | 49 | D-TGCT | 1.0 | THA | Denosumab | No | 0 | N/A | N/A | - | N/A |
|  | 4 | F | 46 | D-TGCT | 1.6 | Arthroscopic synovectomy | Cryotherapy | No | 0 | N/A | N/A | - | N/A |
|  | 5 | F | 16 | D-TGCT | 21.3 | Open synovectomy | No | No | 1 | 0.5 | Arthroscopische synovectomie + radiotherapie | yes | 22 |
|  | 6 | F | 22 | D-TGCT | 1.2 | Arthroscopic synovectomy | No | yes (Avasculaire necrose) | 0 | N/A | N/A | yes | 1.2 |
|  | 7 | F | 31 | L-TGCT | 1.2 | Open synovectomy | No | No | 0 | N/A | N/A | - | N/A |
|  | 8 | F | 28 | D-TGCT | 2.0 | Conservative | No | No | 0 | N/A | N/A |  | N/A |
|  | 9 | F | 38 | L-TGCT | 1.3 | Open synovectomy | No | No | 0 | N/A | N/A | - | N/A |
|  | 10 | F | 17 | L-TGCT | 2.1 | Open synovectomy | No | No | 0 | N/A | N/A | - | N/A |
|  | 11 | M | 38 | D-TGCT | 14.2 | THA | No | No | 0 | N/A | N/A | - | N/A |
|  | 12 | M | 11 | D-TGCT | 9.7 | Open synovectomy | No | No | 0 | N/A | N/A | - | N/A |
|  | 13 | F | 42 | L-TGCT | 0.1 | Conservative | No | No | 0 | N/A | N/A | - | N/A |
|  | 14 | M | 41 | D-TGCT | 3.2 | THA | No | No | 0 | N/A | N/A | - | N/A |
| RUMC | 15 | F | 44 | D-TGCT | 8.8 | THA | Cryotherapy | yes (dislocation) | 1 | 5.3 | Cup revisie |  | N/A |
|  | 16 | F | 34 | D-TGCT | 11.9 | Open synovectomy | No | No | 1 | 1.5 | THA | yes | 1.5 |
|  | 17 | F | 24 | D-TGCT | 7.9 | Open synovectomy | No | No | 0 | N/A | THA | yes | 0.5 |
|  | 18 | M | 49 | L-TGCT | 10.8 | THA | No | No | 0 | N/A | N/A | - | N/A |
|  | 19 | F | 33 | NR | 9.9 | Open synovectomy | No | No | 0 | N/A | N/A | - | N/A |
|  | 20 | F | 16 | D-TGCT | 17.1 | Open synovectomy | No | No | 2 | 1.5 | radiotherapy | yes | 3.8 |
|  | 21 | F | 29 | D-TGCT | 4.9 | Open synovectomy | Radiotherapy | No | 0 | N/A | N/A | - | N/A |
|  | 22 | M | 46 | D-TGCT | 4.8 | Open synovectomy | No | No | 1 | 0.8 | Open synovectomy + radiotherapy | - | N/A |
|  | 23 | F | 28 | D-TGCT | 4.6 | Open synovectomy | Radiotherapy | No | 0 | N/A | N/A | - | N/A |
