# Supplementary material for: Tenosynovial giant cell tumor of the hip: a systematic review and institutional case series with Meta-analysis of recurrence and patient-reported outcomes
Source: J Bone Oncol. 2026 May 25;58:100769. doi: 10.1016/j.jbo.2026.100769 (PMC13241937; doi:10.1016/j.jbo.2026.100769)
Supplement: Supplementary file 11 — Supplementary material 11 [file mmc11.docx]

| Table 10: Mean PROMIS-CAT outcomes in case series | | | |
| --- | --- | --- | --- |
| PROMIS-CAT domain | **Mean T-score (sd)** | **Median T-score** | **Range** |
| Pain interference | 63.4 9 (11.1) | 63.5 | 39 - 76.0 |
| Physical function | 49.2 (11.2) | 48.2 | 23 - 61.7 |
| Fatigue | 49.11 (9.3) | 51 | 33 - 64 |
| Ability to participate in social roles and activities | 44.8 (13.4) | 51 | 29 - 68 |
| Depression | 51.8 (7.8) | 54.3 | 39.6 - 62 |
| Anxiety | 52.8 (8.3) | 53.7 | 38.9 - 63 |

PROMIS-cat = Patient Reported Outcome Measures – Computer Adaptive Test
